# Prolapsus Ani

**Published:** 1879-05

**Authors:** 


Prolapsus Ani.—A boy aged six years was brought into the clinic, suffering from this affection. It had troubled him for two years, but until within the last month the gut only came down at the time of the stools. For the last four weeks it had been down constantly, and neither the mother or her family physician had been able to replace it. In connection with the case Prof. Langenbeck said: "In this disease I have been able to cure *every case* in which I have instituted the treatment I shall adopt in this one, where the patient has been above three years of age.

"Why I have not been equally successful in those of more tender years, I cannot tell, but am disposed to attribute my failures to the fact of younger children being less manageable, and their pressing down efforts when they cry."

His treatment consists in the injection of ergotine into the cellular tissue beside the rectum. He passes the hypodermic syringe parallel with the gut to the depth of about three centimeters, and injects about 20 minims of the solution into each point, usually making the injection at two or three places around the sphincter.

As the mucous membrane of the prolapsed gut in this case was very relaxed and superabundant, he made three parallel incisions through it, about four centimeters in length, with the thermo-cautery, with a view of producing a cicatricial growth that would contract the parts when it healed. This last procedure he did not consider essential to the treatment, but it would probably hasten the cure. The gut was now replaced, and the boy put to bed, and the cure was complete, as the bowel never again came down.

I have been most highly pleased to witness Prof. Langenbeck's operations upon the uterus, and in listening to his lectures upon this subject, not only on account of his wide range of personal experience, but also on account of the fact that I look upon him as a man who is the least influenced by any special hobby of any man that I have ever seen operate on that organ.

